# Ketosis Onset Type 2 Diabetes Had Better Islet **β**-Cell Function and More Serious Insulin Resistance

**DOI:** 10.1155/2014/510643

**Published:** 2014-04-13

**Authors:** Hongyun Lu, Fang Hu, Yingjuan Zeng, Lingling Zou, Shunkui Luo, Ying Sun, Hong Liu, Liao Sun

**Affiliations:** Department of Endocrinology & Metabolism, The Fifth Affiliated Hospital of Sun Yat-sen University, Zhuhai, Guangdong 519000, China

## Abstract

Diabetic ketosis had been identified as a characteristic of type 1 diabetes mellitus (T1DM), but now emerging evidence has identified that they were diagnosed as T2DM after long time follow up. This case control study was aimed at comparing the clinical characteristic, *β*-cell function, and insulin resistance of ketosis and nonketotic onset T2DM and providing evidence for treatment selection. 140 cases of newly diagnosed T2DM patients were divided into ketosis (62 cases) and nonketotic onset group (78 cases). After correction of hyperglycemia and ketosis with insulin therapy, plasma C-peptide concentrations were measured at 0, 0.5, 1, 2, and 3 hours after 75 g glucose oral administration. Area under the curve (AUC) of C-peptide was calculated. Homoeostasis model assessment was used to estimate basal *β*-cell function (HOMA-*β*) and insulin resistance (HOMA-IR). Our results showed that ketosis onset group had higher prevalence of nonalcoholic fatty liver disease (NAFLD) than nonketotic group (*P* = 0.04). Ketosis onset group had increased plasma C-peptide levels at 0 h, 0.5 h, and 3 h and higher AUC_0–0.5_, AUC_0–1_, AUC_0–3_ (*P* < 0.05). Moreover, this group also had higher HOMA-*β* and HOMA-IR than nonketotic group (*P* < 0.05). From these data, we concluded that ketosis onset T2DM had better islet *β*-cell function and more serious insulin resistance than nonketotic onset T2DM.

## 1. Introduction

The presence of ketosis was formerly considered as an important sign of type 1 or insulin-dependent diabetes, but now emerging evidence had indicated that ketosis can happen in type 2 diabetic (T2DM) patients even more than type 1 diabetic mellitus (T1DM). Most them were diagnosed as type 2 diabetes, who had good glucose control, did not need insulin treatment later during long time followup, and were lacking autoimmune markers of *β*-cell destruction [1–3]. Ketosis onset T2DM was considered as an atypical form of diabetes which was first reported in subjects of African origin. It was characterized by obesity, unprovoked ketoacidosis, reversible *β*-cell dysfunctions, and near-normoglycemic remission [4], but now it is widely recognized in several ethnical populations [1, 2, 5]. Some studies have compared islet *β*-cell function among those groups with typical T2DM or T1DM or nondiabetic subjects, but they have not obtained uniform conclusions so far [1–3, 6]. Gosmanov et al. [6] showed that patients with ketosis-prone diabetes displayed a pattern of insulin secretion similar to that of patients with ketosis-resistant T2DM and obese nondiabetic subjects after hyperglycemia remission, but other researches have reported that non-insulin-dependent ketosis-prone T2DM showed lower C-peptide than that of typical T2DM after correction of hyperglycemia and ketosis [4]. The conclusions were controversial.

Furthermore, there has no systematical studies which compared islet *β*-cell function between ketosis and nonketotic onset T2DM patients. Therefore, we designed this case control study to compare the islet *β*-cell function and insulin resistance between ketosis onset and nonketotic onset T2DM patients. Several indicators were used for assessment of islet *β*-cell function, such as HOMA-*β*, which represented basal *β*-cell function; AUC_0–0.5  _ (the area under the curve, AUC) of 0 h to 0.5 h C-peptide and AUC_0–1_ (AUC of 0 h to 1 h C-peptide) which represented the acute insulin response; and AUC_0–3_ which indicated whole *β*-cell capacity. In addition, HOMA-IR was calculated to compare their insulin resistance. We also assessed the metabolic characteristics of two groups.

## 2. Methods

### 2.1. Subjects

From January 2008 to June 2013, a total of 140 cases of newly diagnosed T2DM patients, aged 16~68 years, admitted to our department for medical management were recruited. Diabetes was diagnosed according to WHO diagnostic criteria (1999) [7]. T2DM was diagnosed by clinical characteristics, islet *β*-cell function, and autoimmune diabetic antibodies. In order to exclude the impact of diabetic duration on islet function, typical symptoms of diabetes were lasting for less than one year. Patients whose plasma glucose levels were above 250 mg/dL (13.9 mmol/L) and urine ketone body were positive (above 1+) were diagnosed as diabetic ketosis. With the exception of diabetes, none had evidence of other diseases or were taking agents known to affect carbohydrate metabolism. Patients with obvious precipitating causes for the development of ketosis (such as stress, infection, or trauma) were excluded. Islet cell antibodies including GAD-Ab, IAA, and ICA determined on initial admission were negative in all patients.

### 2.2. Clinical Data Collection

All patients received physical and biochemical examinations after admission to the hospital. Weight (without shoes and in light outdoor clothing) and height were measured. Body mass index (BMI, kg/m^2^) was calculated by dividing weight (kg) by height squared (m^2^). Blood pressure was measured with the Riva-Rocci sphygmomanometer. All venous blood samples were taken in the morning following an overnight fasting for at least 10 hours. Serum total cholesterol, triglyceride, low-density lipoprotein cholesterol (LDL-cholesterol) and high-density lipoprotein cholesterol (HDL-cholesterol), alanine aminotransferase (ALT), and aspartate aminotransferase (AST) were measured using Beckman Biochemical Analyzer (DXC800, USA). HbA1c was measured by high performance liquid chromatography with an automated biochemistry analyzer (Roche, Switzerland). Urine acetone bodies were measured by chemical analysis. Hepatic ultrasonography scanning was performed on all subjects after an overnight fast by assigned and experienced radiologists who were blinded to subjects' details. Nonalcoholic fatty liver disease (NAFLD) was defined by liver ultrasonographic scanning and diagnosed according to the standard set by the Chinese Association of Medicine in 2010 [8], excluding viral hepatitis in nondrinkers.

### 2.3. Islet ***β***-Cell Function and Insulin Resistance Assessment

All patients were treated with insulin intensive therapy on the basis of diet control and diabetic education. Insulin glargine (Sanofi-Aventis) at bedtime and premeal insulin aspart (Novo Nordisk) were used in this study. Initial insulin doses were 0.4-0.5 IU/kg and total daily doses were divided with 50% of bolus and 50% of basal injection. The doses were titrated every day according to the capillary blood glucose in order to reach the glyceamic goal which was defined as fasting blood glucose (FBG) less than 7.0 mmol/L and postprandial blood glucose (PBG) less than 11.1 mmol/L. After correction of ketosis and/or hyperglycemia situation and urine acetone bodies became negative in three consecutive days, evaluation of *β*-cell function and insulin resistance was performed. At one day before testing, insulin treatment was stopped, and 75 g of glucose was orally administered in the morning of the next day. The blood glucose and C-peptide were measured at 0, 30, 60, 120, and 180 min after glucose administration. Plasma C-peptide was determined with a chemiluminescence immunoassays kit (Abbott, Spain). Its interbatch and intrabatch were 1.6% and 2.1%, respectively. Area under the curve (AUC) of C-peptide release test was calculated using the trapezoidal rule. Homoeostasis model assessment was used to estimate basal *β*-cell function (HOMA-*β*) and insulin resistance (HOMA-IR) [3]. The following equations were used to calculate *β*-cell function and insulin resistance: HOMA-*β* = Fasting  C-Peptide × 0.27/(Fasting Plasma Glucose-3.5) and HOMA-IR = 1.5 + Fasting Plasma Glucose × Fasting C-Peptide/2800.

### 2.4. Statistical Analysis

Data were analyzed with the SPSS 17.0 software (SPSS Inc., Chicago, USA). Normally distributed and continuous variables (age, BMI, HbA1c, total cholesterol, LDL-cholesterol, HDL-cholesterol, ALT, and AST) were presented as mean ± SD and analyzed with ANOVA, and nonnormally distributed variables (triglycerides, C-peptide, HOMA-*β*, and HOMA-IR) were expressed as median (IQR) and had been log-transformed into analysis. A Kruskal-Wallis H or Friedman test was used to analyze the nonnormally distributed variables (sex and fatty liver). In all statistical tests, *P* values <0.05 were considered statistically significant.

## 3. Results

### 3.1. Comparison of Clinical Characteristics in Two Groups

The clinical characteristics and metabolic parameters of ketosis onset and nonketotic onset diabetic patients were shown in Table 1. 140 cases of newly diagnosed T2DM without islet-associated autoantibodies were recruited. The mean age was 46.03 ± 3.89 years and the mean BMI was 24.65 ± 2.08 kg/m^2^. There were 62 subjects with ketosis onset and 78 with nonketotic T2DM according to their urine ketone body. In all subjects, male patients (69.3%) were more than female ones. There was no significant difference between two groups regarding other clinical and biochemical parameters, including years, sex, BMI, HbA1c, ALT, AST, and lipid profile (all *P* > 0.05). At remission, ketosis onset and nonketotic group had similar FBG (6.87 ± 0.25 versus 6.76 ± 0.17 mmol/L, *P* = 0.72). The mean time to achieve hyperglycemia remission in ketosis onset group was 5.88 ± 1.45 days and 5.33 ± 1.16 days in nonketotic group (*P* = 0.51). But ketosis onset group had higher percentage of NAFLD than that of nonketotic onset group (58.1% versus 39.7%, *P* = 0.04).

### 3.2. Comparison of  Islet ***β***-Cell Function and Insulin Resistance in Two Groups

**Figure 1:**
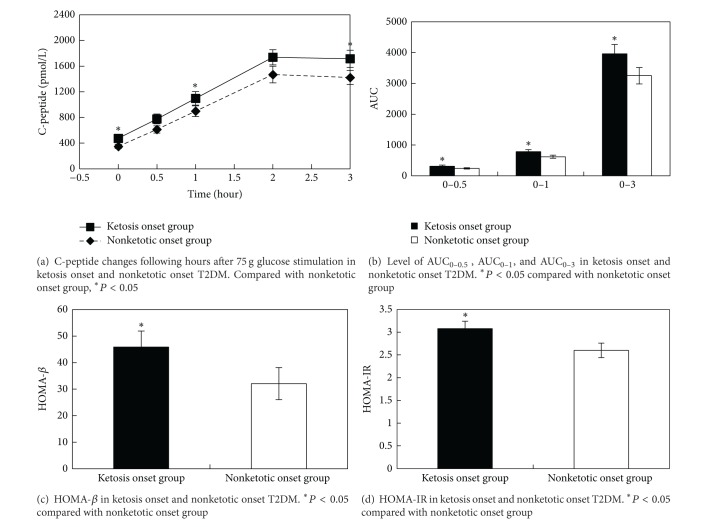
Comparisons of islet *β*-cell function and insulin resistance between two groups.

The characteristics of islet *β*-cell function and insulin resistance were shown in Figure 1. Plasma fasting C-peptide and HOMA-*β* were used to assess basal *β*-cell function. We found that fasting C-peptide level was higher in ketosis onset group than nonketotic group (Figure 1(a)) [475.87(406) versus 348.15(283), *P* = 0.02]. AUC_0–0.5  _ and AUC_0–1_, which represent the acute insulin response, were also higher in ketosis onset group than those in nonketotic group (*P* < 0.05) (Figure 1(b)). The AUC_0–3_, which indicates whole *β*-cell capacity, was also higher in ketosis onset group than that in nonketotic onset group [3960.66(2349.75) versus 3248.55(2361.69), *P* = 0.02]. HOMA-*β* was also higher in ketosis onset group than that in nonketotic group (Figure 1(c)) [45.86(6.05) versus 32.08(2.77), *P* = 0.03]. HOMA-IR, which shows insulin resistance, was also higher in ketosis onset group than that in nonketotic onset group (Figure 1(d)) [3.08(1.25) versus 2.60(1.08), *P* = 0.02].

## 4. Discussion

Ketosis onset diabetes is increasingly recognized by featuring with an emerging syndrome of obesity, unprovoked ketoacidosis, reversible *β*-cell dysfunction, and near-normoglycemic remission [4, 5]. In our previous research, we found that diabetic ketosis was occurred more frequently in T2DM than T1DM on admission; they often showed obvious polyphagia, polydipsia, polyuria, and weight loss in a short time, but better islet *β*-cell function in long time followup and lack of autoimmune diabetic antibodies.

Previous study had found that ketosis onset group had a strong predominance in male patients [9]; in our case control study, we found that ketosis onset group patients had lower predominance of male (66.1% versus 71.8%), but there was no significant difference (*P* = 0.58). We also found that AUC_0–0.5_, AUC_0–1_, and AUC_0–3_ were all higher in ketosis onset group than those of nonketotic group. It indicated that ketosis onset group had better acute insulin response and *β*-cell capacity than nonketotic group. Moreover, ketosis onset group had more serious insulin resistance. These results were consistent with the previous report by Mauvais-Jarvis et al. [4]. They measured insulin secretion (glucagon-stimulated C-peptide) and insulin action (short intravenous insulin tolerance test) in T2DM, T1DM, and ketosis-prone diabetes (KPD) groups during a 10-year followup. The result showed that triglyceride levels were similar and insulin-dependent KPD group had more serious insulin resistance than T2DM. But other studies did not get the same results. Gosmanov et al. [6] found that AIR (acute insulin response) and FPIR (first-phase insulin release) to arginine stimulation, as well as changes in insulin, C-peptide, and the C-peptide-to-glucose ratio during a 20 h dextrose infusion, were similar among KPD, T2DM, and obese control subject. We speculate that different ethnical population may have different insulin secretion characteristics.

We also found that ketosis onset group had higher percentage of NAFLD than that of nonketotic onset group (58.1% versus 39.7%, *P* = 0.04), which was not reported in other studies. Insulin resistance and fatty liver play an interaction in the development of  T2DM [10, 11]. There was strong evidence to support the fact that fatty liver affected insulin signaling in insulin-responsive tissues by producing humoral factors, such as FGF21 [12], fetuin-A [13, 14], and retinol binding protein 4 (RBP4) [15]. In addition, Zhou et al. [16] found that the KPD patients were more likely to be accompanied with fatty liver (10.1%) compared with those with T1DM. Choukem et al. [17] observed the triad hepatic, adipose tissue, and skeletal muscle insulin resistance in patients with KPD during near-normoglycemic remission. We can conclude that fatty liver is a character of KPD. Whether fatty liver plays a crucial role in the development of ketosis onset T2DM remains unclear and more studies are needed.

Although ketosis onset diabetes is sometimes called KPD, both present with DKA or unprovoked ketosis, KPD is defined as a widespread, emerging, heterogeneous syndrome which does not necessarily have the typical phenotype of autoimmune T1DM [18, 19], but ketosis onset diabetic patients present with ketosis or ketoacidosis without known diabetes [20, 21]. So we select this group to study.

The pathogenesis of ketosis onset diabetes still remains unclear. It comprises reversible insulin secretion, defect partially, and some degree of insulin resistance. Recently many studies focused on KPD have been done to unravel the mechanisms of this disease. KPD was divided into four subgroups according to the presence of glutamic acid decarboxylase (GAD) 65, GAD67, or IA-2 autoantibodies (A^+^ or A^−^) and *β*-cell functional reserve (*β* ^+^ or *β* ^−^). The group distribution was A^+^ *β* ^−^, A^−^ *β* ^+^, A^−^ *β* ^−^, and A^+^ *β* ^+^ [22, 23]. Choukem et al. showed that *β*- and *α*-cell dysfunctions both contribute to the pathophysiology of KPD; in addition, KPD displays a defect in *β*-cell sensitivity to glucose and has reduced *β*-cell mass [24]. Umpierrez et al. demonstrated that hyperglycemia, but not lipotoxicity, played the crucial role in the pathogenesis of KPD in obese African patients [25]. Gene variation may be another important factor involved in it. PAX4 (paired box4) is a transcription factor essential for the development of insulin-producing pancreatic *β*-cells. Mauvais-Jarvis et al. had found gene polymorphism of PAX4 (Arg121Trp and Arg133Trp) in Japanese and west Africans origin [26]. Sobngwi et al. had described a high prevalence of glucose-6-phosphate-dehydrogenase (G6PD) deficiency without gene mutation in these patients [27]; then Choukem et al. reported that polymorphism of Arg585Gln in SREBF-1 was not associated with the KPD phenotype [28].

Some limitation of this study should be addressed. Firstly, the diagnosis of ketosis was based on patients' positive urine ketone body but not on plasma ketone body. We all know plasma ketone body is more sensitive and specific to diagnose diabetic ketosis, but because this study was a retrospective study, our institute had not conducted plasma ketone body detection in early time, so we selected urine ketone body as surrogate marker, which was widely used and recognized in clinical practice. Secondly, because ketosis often occurred in the state of high glucose, we did not test islet function on initial admission. Whether ketosis onset group had better islet function on initial admission or stronger ability of recovery than nonketotic group remained unknown. Thirdly, in order to observe their *β*-cell function, long time following up and prospective studies in this area are needed.

## 5. Conclusion

This study has notable strengths. We found that ketosis onset T2DM had better islet *β*-cell function and more serious insulin resistance than nonketotic onset T2DM.

## Figures and Tables

**Table 1:** Clinical and biochemical parameters of subjects.

Variable	Ketosis onset T2DM	Nonketotic onset T2DM	*P*
*n*	62	78	
Age (years)	44.84 ± 1.15	46.98 ± 1.03	0.17
Sex (*n*)			
Male/female	41/21	56/22	0.58
BMI (kg/m^2^)	25.01 ± 0.52	24.37 ± 0.37	0.30
SBP (mmHg)	123.24 ± 2.14	126.78 ± 2.39	0.30
DBP (mmHg)	82.98 ± 1.43	82.12 ± 1.45	0.69
HbA1c (%)	11.02 ± 2.73	11.84 ± 2.75	0.08
Cholesterol (mmol/L)			
Total	5.37 ± 0.14	5.65 ± 0.17	0.13
HDL-C	1.08 ± 0.06	1.12 ± 0.03	0.26
LDL-C	3.29 ± 0.14	3.44 ± 0.12	0.88
Triglycerides (mmol/L)	2.67 (2.16)	2.07 (1.21)	0.35
ALT (U/L)	30.48 ± 2.81	31.85 ± 3.93	0.46
AST (U/L)	23.82 ± 1.62	23.91 ± 2.21	0.25
Fatty liver (*n*)			
Yes/no	36/26	31/47	0.04

Normally distributed and continuous variables (age, BMI, HbA1c, total cholesterol, LDL-cholesterol, HDL-cholesterol, ALT, and AST) were presented as mean ± SD and analyzed with ANOVA, nonnormally distributed variables (triglycerides) were expressed as median (IQR) and have been log-transformed into analysis. A Kruskal-Wallis H or Friedman test was used to analyze the nonnormally distributed variables (sex and fatty liver). In all statistical tests, *P* values < 0.05 were considered statistically significant.

BMI: body mass index; SBP: systolic blood pressure; DBP: diastolic blood pressure; HbA1c: hemoglobin A1c; HDL-C: high-density lipoprotein cholesterol; LDL-C: low-density lipoprotein cholesterol; ALT: alanine aminotransferase; AST: aspartate aminotransferase.
